# Chromogranin a and pancreatic polypeptide are not suitable for the screening of pancreatic neuroendocrine tumors in MEN1 - a long-term follow-up study

**DOI:** 10.1007/s12020-025-04291-y

**Published:** 2025-06-02

**Authors:** Iiro Kostiainen, Susanna Majala, Jukka Schildt, Helka Parviainen, Saila Kauhanen, Niina Matikainen, Eeva M. Ryhänen, Camilla Schalin-Jäntti

**Affiliations:** 1 Endocrinology, Abdominal Center, Helsinki University Hospital and the University of Helsinki, Helsinki, Finland; 2 Department of Surgery, Division of Digestive Surgery and Urology, Turku University Hospital and University of Turku, Turku, Finland; 3 Turku PET Centre, Turku University Hospital, Turku, Finland; 4 HUS Medical Imaging Center, Department of Clinical Physiology and Nuclear Medicine, Helsinki University Hospital and the University of Helsinki, Helsinki, Finland; 5 HUS Medical Imaging Center, Department of Radiology, Helsinki University Hospital and the University of Helsinki, Helsinki, Finland; 6 Department of Radiology, Vaasa Central Hospital, Wellbeing Services County of Ostrobothnia, Vaasa, Finland

**Keywords:** MEN1, Chromogranin A, Pancreatic polypeptide, Pancreatic neuroendocrine tumor, Somatostatin receptor PET/CT

## Abstract

**Purpose:**

In patients with multiple endocrine neoplasia type 1 (MEN1) followed up at ENETS centers of Excellence, chromogranin A (CgA) and pancreatic polypeptide (PP) are widely used screening tools for pancreatic neuroendocrine tumors (panNETs). Previous studies have demonstrated conflicting results regarding their performance in MEN1. This retrospective study aims to bring clarity to the question by investigating a well-characterized MEN1 cohort. We studied the impact of long-term biomarker follow-up on the clinical management of panNETs in MEN1.

**Methods:**

We calculated the sensitivity and specificity and performed ROC analysis of CgA and PP for diagnosing any panNET, ≥20 mm panNET, and metastatic panNET in comparison to imaging reference standard in 58 MEN1 patients. All patients had undergone somatostatin receptor PET/CT and conventional imaging. Longitudinal impact of 10-year annual biomarker measurements on real-life clinical management was analyzed from patient records.

**Results:**

Sensitivity of CgA (*n* = 48) and PP (*n* = 47) for diagnosing any panNET, ≥20 mm panNET, and metastatic panNET was 35%, 30%, and 60 and 23%, 33%, and 0%, respectively. For CgA, the AUC for diagnosing any panNET, ≥20 mm panNET, and metastatic panNET was 0.30 (95% CI 0.09–0.51), 0.49 (95% CI 0.29–0.68), and 0.69 (95% CI 0.42–0.95), respectively. For PP, the AUC for detection of metastatic panNET was 0.28 (95% CI 0.11–0.46). The annual biomarker measurements during 514 patient-years of follow-up did not affect the clinical management of panNETs.

**Conclusion:**

CgA and PP are not helpful in diagnosing panNETs in MEN1. It is time to revise the surveillance protocols in practice.

## Introduction

Pancreatic neuroendocrine tumors (panNETs) are frequently observed among patients with type 1 multiple endocrine neoplasia (MEN1) and also the leading cause of death for these patients [1, 2]. Consequently, accurate diagnosis, follow-up, and treatment of panNETs in MEN1 is paramount.

Current international MEN1 guidelines recommend annual biomarker screening of patients with MEN1, including chromogranin A (CgA) and pancreatic polypeptide (PP) screenings for diagnosis and follow-up of patients with gastroenteropancreatic neuroendocrine neoplasms (NENs) [3]. A recent survey of the screening and surveillance practices of patients with MEN1 in ENETS centers of excellence demonstrates frequent screening with CgA and PP in patients with no diagnosis of panNET, with the majority of centers using at least CgA [4].

However, the recommendations of annual CgA and PP screening in this rare hereditary setting can be questioned, as they mirror those recommended for sporadic gastroenteropancreatic NENs, while the actual data on their performance in MEN1 patients with nonfunctioning panNETs is limited [5]. Contemporary evidence of biomarker performance in sporadic panNETs suggests that CgA can differentiate metastatic panNETs from no visible panNET on conventional imaging [6]. However, recent studies on the performance of CgA as a predictor of sporadic nonmetastatic panNETs suggest low sensitivity [7–10]. Reported sensitivity of PP in sporadic nonmetastatic and metastatic panNETs is low [11], although Panzuto et al. [12] found that combining CgA and PP increased the sensitivity for diagnosing panNETs.

Published data on biomarker performance for early detection of panNETs in MEN1 is scarce. Most previous studies have not specifically focused on biomarker performance, but instead, reported biomarker data within the context of broader research questions. These studies are hampered by small patient numbers (*n* = 7–36), lack of standardized pancreatic imaging reference including no description of the actual tumor findings with regards to number and size [5]. There are only two original studies that have focused purely on diagnostic performance of biomarkers in panNETs in patients with MEN1. In their study, de Laat et al. [13] indicated that CgA, PP, and glucagon had poor capability of differentiating patients with and without panNETs when compared to histopathological findings or conventional imaging results. Similarly, Qiu et al. [14] questioned the value of CgA, PP, and glucagon, as well as gastrin, for diagnosing panNETs in MEN1.

In contrast, Sadowski et al. [15] reported that PP correlated with the total number of NET lesions detected on sensitive somatostatin receptor positron emission tomography/computed tomography (SSTR PET/CT) imaging in 26 patients with MEN1. Tirosh et al. [16] noted that CgA correlated with total NET tumor volume assessed by SSTR PET/CT in patients with sporadic panNETs (*n* = 112), while PP correlated with NET tumor volume in the subgroup of hereditary panNETs, including 39 patients with MEN1 and 42 with von Hippel-Lindau disease. These findings raise the question whether the inclusion of SSTR PET/CT data could alter the view on biomarker performance. Based on the results of the recent survey on ENETS centers of excellence demonstrating broad use of biomarker screening [4], further information on biomarker performance could lead to alterations in clinical management even in the most up-to-date providers.

The aim of our study was to assess the performance of CgA and PP in the diagnosis of nonfunctioning panNETs in a well-characterized MEN1 cohort that had undergone sensitive SSTR PET/CT imaging in addition to regular conventional pancreatic imaging. Biomarker performance was assessed at three clinically relevant endpoints, that is, diagnosis of any primary panNET; panNETs ≥ 20 mm in diameter, where surgical intervention was recommended [17]; and metastatic panNET. The secondary aim of our study was to assess the impact of long-term (10-year) annual biomarker measurements on real-life clinical management using longitudinal follow-up data from the electronic patient records.

## Materials and methods

### Study design and population

This retrospective study includes 58 MEN1 patients from the Finnish reference center on rare endocrine conditions (EndoERN FIN consortium) at the Helsinki and Turku University Hospitals, Finland.

**Fig. 1:**
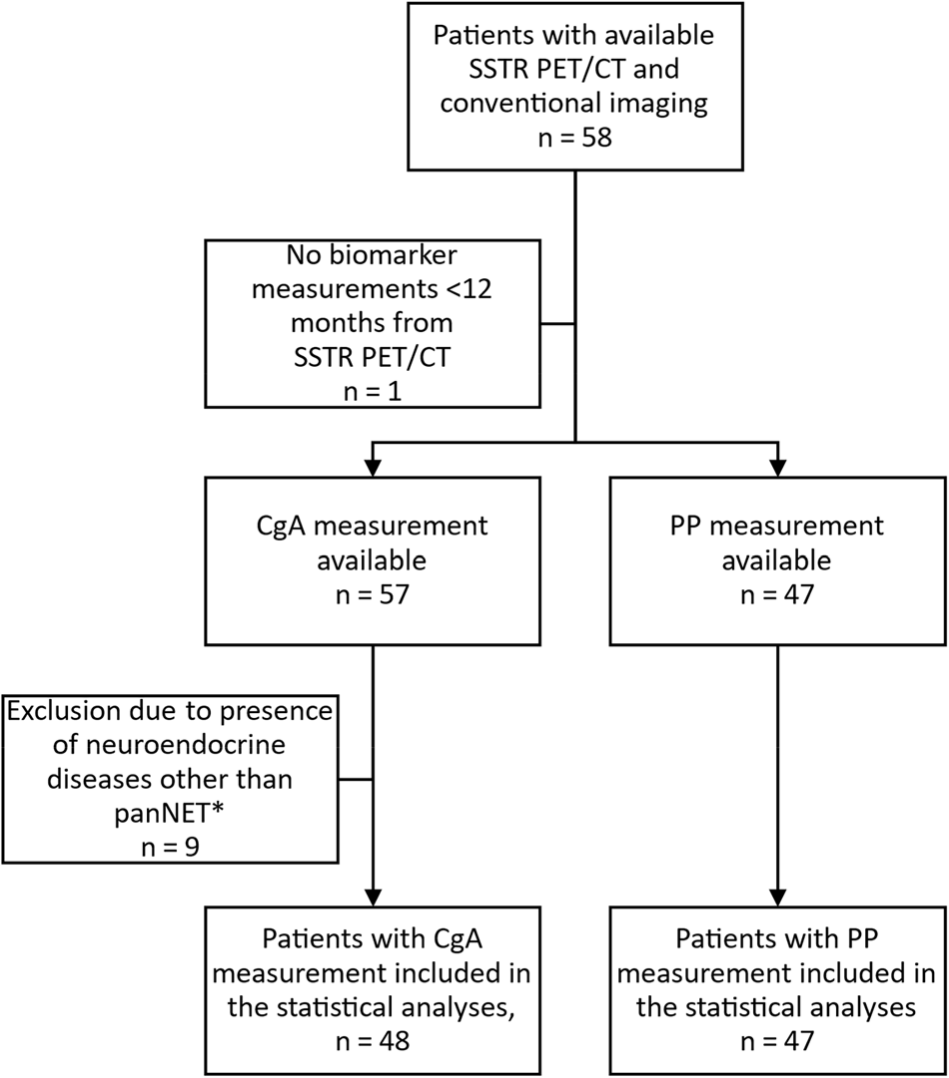
Flowchart of patients included in the analyses comparing biomarker performance to the reference standard. *Lung neuroendocrine tumor (*n* = 5), duodenal neuroendocrine tumor (*n* = 2), mediastinal neuroendocrine carcinoma (*n* = 1), diffuse idiopathic pulmonary neuroendocrine cell hyperplasia (*n* = 1)

We assessed performance of CgA and PP in the diagnosis of panNETs at three clinically relevant endpoints: 1) detection of any panNET, 2) diagnosis of panNETs exceeding 20 mm in diameter (the cut-off for surgery), and 3) diagnosis of metastatic panNET. A flow chart depicting the patients included in the assessment of biomarker performance compared to the panNET imaging reference standard, is shown in Fig. 1. The reference standard for diagnosing any panNET was panNET detected on SSTR PET/CT imaging. The reference standard for diagnosing a panNET ≥ 20 mm in diameter was conventional imaging with MRI or multiphase contrast-enhanced CT; in addition, tumors had to be SSTR PET/CT positive pancreatic lesions. For metastatic disease, the reference standard was metastatic disease detected either with SSTR PET/CT or MRI/CT imaging that was confirmed during further imaging follow-up (median 47 months).

Patients were required to have biomarker measurements within 12 months from the reference SSTR PET/CT images. No interventions that could impact tumor volume (i.e., surgery, peptide receptor radionuclide therapy, initiation of somatostatin analog (SSA), or other systemic antiproliferative treatment) were allowed between biomarker measurements and imaging studies. The presence of concomitant neuroendocrine tumors other than panNETs on imaging was an exclusion criterion for CgA analyses. Local surveillance protocols include, in addition to abdominal imaging, screening of the thoracic region for lung and thymic NETs with thoracic CT every five years. The median temporal difference between biomarker measurements and SSTR PET/CT imaging was 1 month [interquartile range (IQR) 0.3–1.8]. Possible use of SSA and proton pump inhibitor (PPI) at the time of biomarker measurement was assessed from the patient records. All patients routinely received written instructions regarding PPI use before CgA testing, and temporary replacement of PPI with antacids was instructed. For PPI to be considered not in use, a two-week period of restraint prior to testing was required.

In addition, we assessed the impact of annual long-term (>10 years) biomarker screening with CgA and PP from our electronic patient records and whether this led to changes in the management of MEN1 patients in real-life, especially with regards to management of panNETs. All patients from the Helsinki University Hospital were included (*n* = 47). According to the local MEN1 follow-up protocol, biomarkers were measured annually. In patients diagnosed with panNET, conventional imaging was conducted annually. For some patients, especially in the early 2000s, biomarker measurements were performed twice a year. The data evaluated spanned from May 30, 2002, until July 7, 2023, with data collection finalized on July 9, 2023. The total available follow-up was 514 patient years, with a median of 10 years per patient (IQR 5–17, range 2–21).

Patient records were analyzed for possible changes in real-life clinical patient management over time that were attributable to measurements of CgA, PP, or both. Any alteration in clinical management (i.e., initiation of intervention or deviation from standard or preceding follow-up scheme) due to increased biomarker concentrations was considered relevant. The possible relationships between biomarkers and treatment responses in patients with metastasized disease was out of scope of the study and not assessed.

The study was performed as per the Declaration of Helsinki. Authorization to perform this study without individual consent was granted by the institutional review boards of Helsinki University Hospital (HUS/115/2020) and Turku University Hospital (T03/011/21).

### Laboratory measurements

Chromogranin A was measured in two accredited laboratories from fasting plasma samples. CgA was either measured by radioimmunoassay (RIA) or time-resolved amplified cryptate emission (TRACE). Thus, an RIA based assay [upper limit of normal (ULN); 3.0 nmol/l] was used in Turku University Hospital and between June 12, 2013, and March 9, 2021, in the Helsinki University Hospital. Thereafter, CgA was determined by TRACE (ULN; 1.6 nmol/l) in the Helsinki University Hospital. All CgA concentrations used in the current analyses were scaled and displayed based on the ULN. Based on previous research by Choi et al., the correlation between TRACE and RIA-based CgA measurements is high at 0.956 [18]. In quantitative analyses of our study, the majority of CgA measurements were done with RIA (*n* = 47), with only one measurement with TRACE.

For the extended long-term follow-up of patients in the Helsinki University Hospital, additional RIA-based CgA assays were used (ULN; 4.0 nmol/l until January 10, 2010, and ULN 6.0 nmol/l from January 11, 2010, until June 11, 2013).

PP measurements were performed in one accredited laboratory from fasting serum samples using RIA (ULN; 100 pmol/l). The same assay was used throughout the study period.

Glomerular filtration rate (GFR) was calculated with the CKD-EPI equation [19] from creatinine measured synchronously with the biomarkers. As the equation is only applicable to patients aged ≥ 18 years, patients aged < 18 years at biomarker measurement (*n* = 3) were excluded from the analyses.

### Imaging protocols and analysis of imaging data

Detailed imaging protocols and analysis of imaging data are described in our previous study of the same cohort [20]. Size analysis of panNETs was based on MRI or multiphase contrast-enhanced CT. Due to limited accuracy, the low-dose localizing CT of SSTR PET/CT was not used in assessment of panNET diameter.

### Statistical analysis

Statistical analyses were performed using R statistical software (v4.2.1; R Core Team 2022). Data are presented as medians, interquartile ranges, and ranges for continuous variables. The categorical variables are presented as frequencies and proportions. Due to the non-normal distribution of biomarkers, either nonparametric tests or parametric tests with logarithmic transformation were used for the statistical evaluation of continuous variables.

We could not estimate the prevalence of primary panNET at the start of the study to estimate the optimal sample size. However, a post hoc power calculation indicates that based on the occurrence of anatomical panNET (56%), the primary endpoint of our quantitative analyses, the study had over 80% power to reject null hypothesis of AUC ≥ 0.75 in biomarker diagnostic capability when assuming α to be 0.05.

Spearman correlations of biomarkers and different clinical characteristics were calculated. The sensitivity and specificity of biomarkers at diagnostic endpoints were assessed at values exceeding the ULN. For the ROC analysis, models were built with the assumption that higher biomarker concentrations were associated with more advanced panNET disease. An area under the curve (AUC) ≥ 0.80 in ROC was considered indicative of a good diagnostic performance [21]. The reported *P*-values are two-sided, with a *P*-value of < 0.05 considered statistically significant.

## Results

### Patient characteristics

The characteristics of patients included in the analyses of biomarker performance compared to the reference standard for diagnosing panNETs are described in Table 1.

**Table 1:** Characteristics of patients in analyses comparing biomarker performance to the reference standard

Variable	Patients included in CgA analyses (*n* = 48)	Patients included in PP analyses (*n* = 47)
Age at SSTR PET/CT imaging	37 (25–47) [16–68]	38 (28–49) [16–66]
Sex, female/male (n)	23/25 (48%/52%)	24/23 (51%/49%)
PanNET stage		
No panNET (n)	6 (13%)	7 (15%)
PanNET with no metastases (n)	37 (77%)	36 (77%)
Lymph node metastasis of panNET with no other panNET metastases^a^ (n)	3 (6%)	3 (6%)
Other panNET metastases^a^ (n)	2 (4%)	1 (2%)
Patients with a measurable panNET on MRI/CT (n)	27 (56%)	26 (55%)
Diameter of the largest panNET (mm)	14 (9–22) [5–99]	15 (9–23) [5–99]
Previous pancreatic surgery (n)	7 (15%)	8 (17%)
Enucleation (n)	3 (6%)	3 (6%)
Partial resection of pancreas (n)	4 (8%)	3 (6%)
Pancreatectomy (n)	0 (0%)	2 (4%)
Other simultaneous neuroendocrine neoplasia (n)	0 (0%)	5 (11%)
CgA (proportion of ULN)	0.8 (0.7–1.4) [0.3–7]	0.9 (0.7–1.4) [0.3–18]
PP (pmol/l)	56 (22–103) [10–13,125]	47 (20–89) [10–13,125]
GFReEPI (ml/min/1.73 m^2^)	101 (94–115) [44–136]	100 (94–112) [44–136]
Somatostatin analog use^b^ (n)	6 (13%)	6 (13%)
Proton pump inhibitor use^b^ (n)	1 (2%)	1 (2%)

Data are presented as median (interquartile range)/[range] or frequency (proportion), as appropriate

*CgA* chromogranin A, *CT* computed tomography, *GFReEPI* estimated glomerular filtration rate, *MRI* magnetic resonance imaging, *NET* neuroendocrine tumor, *panNET* pancreatic neuroendocrine tumor, *PP* pancreatic polypeptide, *SSTR PET/CT* somatostatin receptor positron emission tomography/computed tomography, *ULN* upper limit of normal

^a^Regardless of the status of primary panNET

^b^In use during laboratory testing. Two-week abstinence prior to testing was required for proton pump inhibitors not to be considered in use

### Relationships between biomarkers and number or size of panNETS

**Fig. 2:**
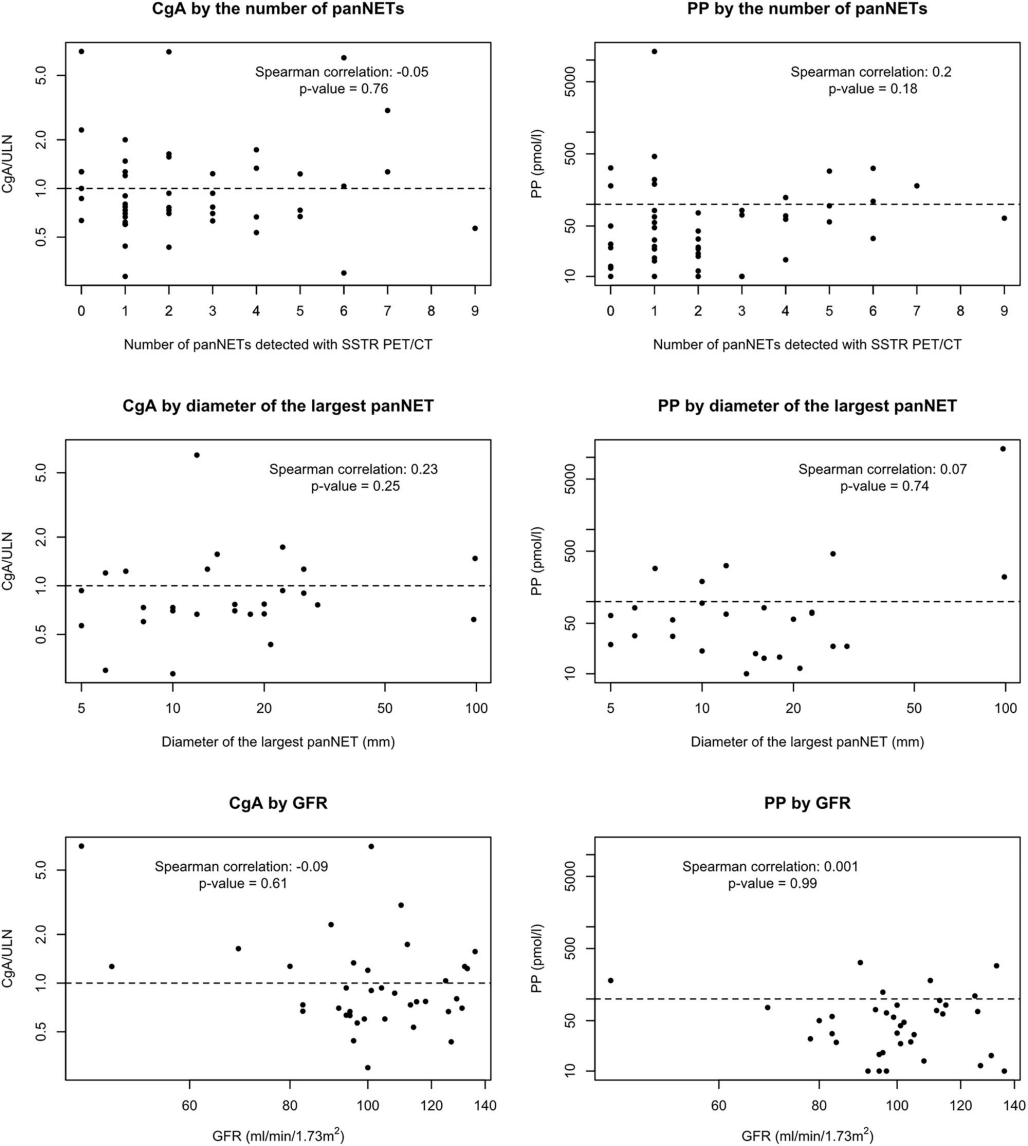
Scatter plots comparing biomarkers with the number of pancreatic neuroendocrine tumors (panNET) detected in somatostatin receptor positron emission tomography/computed tomography (SSTR PET/CT), size of the largest panNET and glomerular filtration rate (GFR). Horizontal dotted Line denotes the upper limit of normal (ULN) for biomarker concentration

The scatter plots demonstrating the distribution of CgA and PP concentrations according to number and size of panNETs and GFR are shown in Fig. 2. Neither CgA nor PP correlated with the number of panNETs, diameter of largest panNET, or GFR. Chromogranin A or PP did not correlate with age or gender. When adjusted for presence of lymph node or other metastasis, no significant correlations were noted between biomarkers and SSA use or previous pancreatic surgery (including resection or total pancreatectomy, but not enucleation).

### Biomarker concentrations according to panNET stage

**Fig. 3:**
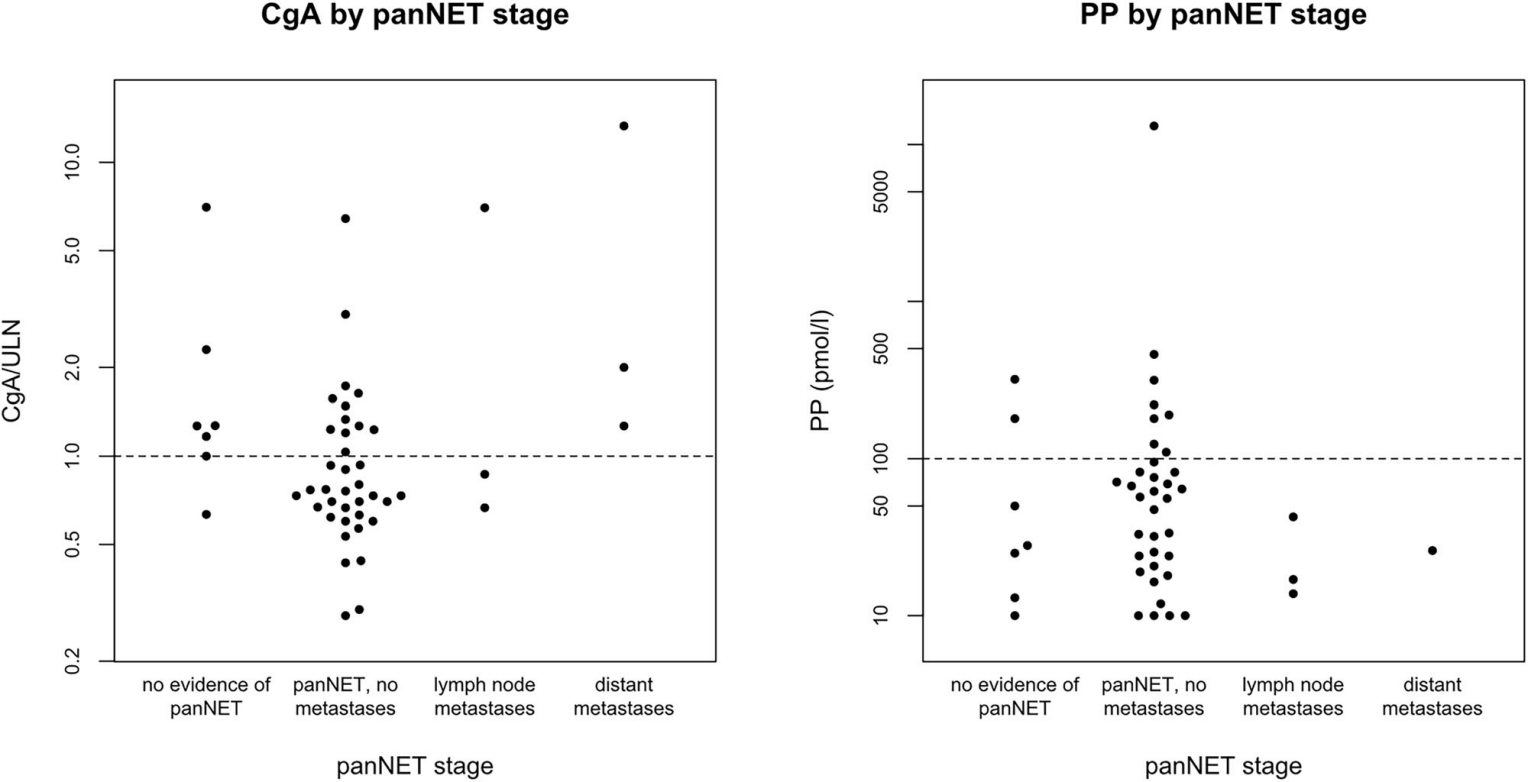
Dot plots comparing levels of biomarkers with panNET stage. Horizontal dotted line denotes upper limit of normal (ULN) for biomarker concentration

The dot plot of biomarker concentrations according to panNET stage is shown in Fig. 3.

### Sensitivity and specificity of CgA and PP for detection of any PanNET, ≥ 20 mm panNET, and metastatic panNET

The sensitivity and specificity of biomarkers for diagnosing panNETs are given in Table 2. The sensitivity for diagnosing any panNETs restricted to the pancreas was poor ( ≤ 35%) for both biomarkers. For the detection of metastasized panNET, the sensitivity and specificity of CgA were 60 and 63%, respectively.

**Table 2:** Observed sensitivity and specificity of biomarkers compared to the reference standard for clinically relevant diagnostic endpoints

Diagnostic endpoint	Biomarker	Sensitivity	Specificity
Detection of primary panNET	CgA	35% (14/40)	38% (3/8)
	PP	23% (9/39)	75% (6/8)
Detection of panNET with diameter of ≥ 20 mm	CgA	30% (3/10)	58% (22/38)
	PP	33% (3/9)	79% (30/38)
Detection of metastatic panNET^a^	CgA	60% (3/5)	63% (27/43)
	PP	0% (0/4)	74% (32/43)

Biomarker concentration exceeding the upper limit of normal was considered positive

*CgA* chromogranin A, *panNET* pancreatic neuroendocrine tumor, *PP* pancreatic polypeptide

^a^Lymph node or other metastases of panNET, regardless of the status of primary panNET

### Receiver operator characteristics of CgA and PP for detection of any panNET, ≥ 20 mm panNET, and metastatic panNET

**Fig. 4:**
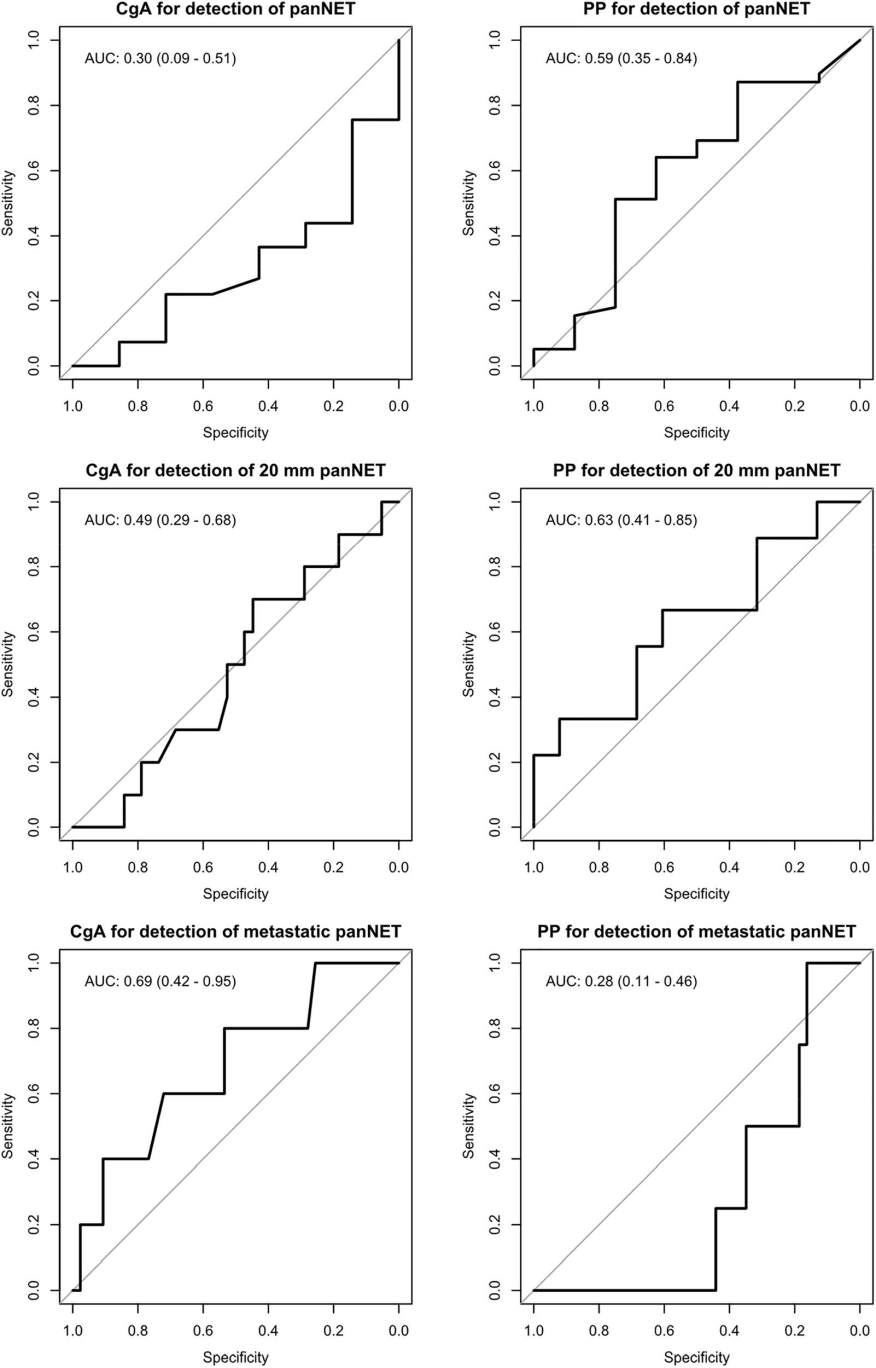
Receiver operator characteristics (ROC) analysis of biomarker performance for clinically relevant endpoints. 95% CI of the area under the curve (AUC) in parentheses

The results of the ROC analysis are shown in Fig. 4.

For CgA, AUC for diagnosis of any primary panNET was 0.30 (95% CI 0.09–0.51). The AUC for the detection of ≥ 20 mm panNET was 0.49 (0.29–0.68). When acceptable performance was set at AUC of ≥ 0.80, subpar performance reached statistical significance. For the detection of metastatic disease, AUC was 0.69 (0.42–0.95), which was not statistically significant.

The ROC analyses for CgA performance were also assessed after excluding the additional confounding factors (PPI and SSA use, and GFR < 60 ml/min/m^2^). The AUCs for CgA for the diagnosis of primary panNET, detection of ≥ 20 mm panNET, and metastatic panNET were 0.24 (0.08–0.41), 0.51 (0.30–0.73), and 0.84 (0.56–1.00), respectively.

For PP, the observed performance in the ROC analysis was poor at all endpoints (Fig. 4). The 95% CI of AUC was below the ≥ 0.80 threshold only for the detection of metastatic disease 0.28 (0.11–0.46).

### Assessment of patients with increased CgA and no detectable panNET on imaging

Four of the six patients with no evidence of panNET on conventional or SSTR PET/CT imaging had increased CgA concentrations, i.e. were false positive and analyzed for possible underlying factors. One of them had chronic renal failure (GFR 44 ml/min/m^2^). Another was diagnosed with pancreatic adenocarcinoma and underwent pancreaticoduodenectomy (the tail and body of the pancreas had been resected previously because of panNETs), and CgA normalized after surgery. In addition to pancreatic adenocarcinoma, hyperplasia of neuroendocrine tissue of pancreas and numerous small (<2 mm) panNETs were discovered through histopathology. Of the two remaining patients, one had transient CgA elevation. No specific explanations for the increased CgA concentrations could be found in the patient records.

### Assessment of the real-life clinical impact of long-term, annual biomarker follow-up

During total follow-up of 514 patient years, biomarker measurements led to changes in clinical management on eleven occasions, only three of which led to any other alteration than controlling i.e., repeating, the biomarker measurement.

In the first case, increased PP (46% rise from the preceding concentration to 1.2 times ULN) prompted imaging with SSTR PET/CT 12 months ahead of schedule. The SSTR PET/CT demonstrated four small panNETs, only one of which (8 mm) was visible on MRI. The increase in PP was transient during 3 years of follow-up and PP remained normal, while conventional imaging demonstrated no further tumor growth. In the second case, both a gradual increase in CgA and a sudden elevation of PP (70% rise to 1.9 times ULN) prompted additional SSTR PET/CT imaging, but no NETs were identified. During more than 6 years of follow-up, CgA and PP remained persistently elevated with no evidence of NEN on conventional imaging. In the third case, increased CgA (1.1 times ULN) in a patient previously treated for lung NET prompted a repeat measurement of CgA 6 months later. As CgA increased further (48% rise, to 1.6 times ULN), SSTR PET/CT was conducted, the patient was diagnosed with recurrent inoperable lung NET, and SSA treatment was initiated, leading to a single significant alteration in clinical management in this patient population.

On eight occasions, either biomarker elevation (*n* = 3), laboratory test failure (*n* = 3), or patient nonadherence to test prerequisites (*n* = 2) led to additional biomarker testing, with no further impact on clinical management.

## Discussion

In the present study, we demonstrate in a MEN1 population that had undergone sensitive SSTR PET/CT imaging in addition to conventional imaging follow-up of panNETs, that CgA and PP lack diagnostic accuracy for panNETs. Biomarker sensitivity was low compared to imaging standard in all categories. Biomarker screening did not detect any new panNETs and was not helpful in diagnosing clinically significant panNETs ≥ 20 mm or detecting metastatic disease. The performance of biomarkers was equally poor in the ROC analysis, as CgA was unable to distinguish between patients with panNETs or those with panNETs ≥ 20 mm. The observed performance of PP was also poor, but it did not reach statistical significance except in the detection of metastatic disease.

As some studies on sporadic panNETs have demonstrated that CgA and PP can predict metastatic disease [11, 12, 22], it is possible that repeat biomarker measurement could be useful for the early detection of metastatic disease in MEN1 as well. Our study did not demonstrate such ability, as only one-third of the patients with nodal involvement on SSTR PET/CT had elevated levels of CgA, while none had increased PP. Similarly, Mirkin et al. [23], noted that nodal involvement in histopathological analysis of sporadic panNETs was not associated with elevated CgA concentration. Not even long-term, real-life, repeat biomarker measurement over 10 years yielded any results, as no clinically significant biomarker-attributable interventions (such as surgery or other treatment) for panNETs occurred during the 514 follow-up years in patients with MEN1. Only one case of increased PP concentration—which was later confirmed to be transient—led to an earlier diagnosis of small panNETs, as this patient underwent SSTR PET/CT 12 months ahead of the schedule. Beyond the scope of panNETs, in one patient, increased CgA led to earlier detection of recurrent inoperable lung NET.

Most previous data on the performance of biomarkers in the detection of panNETs in MEN1 is derived from only two high quality original studies by de Laat et al. [13] and Qiu et al. [14]. Other previous original studies are hampered by small MEN1 patient numbers, lack genetic confirmation of MEN1 diagnosis, and include mixed NET patient populations [24–27]. Importantly, they lack a standardized pancreatic imaging reference and actual pancreatic imaging results are not reported [24, 28], or a relevant control group is not included [26]. Compared to the two previous studies by de Laat et al. [13] and Qiu et al. [14], in addition to conventional pancreatic imaging, our study includes a uniform imaging reference standard based on sensitive SSTR PET/CT, which enables the detection of even very small panNETs, and demonstrates poor biomarker performance for the detection of clinically significant > 20 mm panNETs. Another strength of the study is the real-life long-term data: not even annual biomarker surveillance for more than 10 years was helpful and did not alter patient management.

Of note, a recent survey in 56 ENETS centers of excellence demonstrated that a majority, i.e. 69% of the centers still use CgA and 38% PP in the screening of duodeno-pancreatic NETs in patients with MEN1 [4]. It is evident that not even ENETS centers of excellence have implemented the results by de Laat et al. [13] and Qiu et al. [14], published in 2013 and 2016. There is an unmet need for additional high quality original studies on the usefulness of CgA and PP screening in MEN1, such as our study, to provide evidence for changing the MEN1 follow-up protocols in practice.

Although CgA and PP are widely available and practical screening tools, our study and previous reviews [5, 29] do not support the inclusion of CgA and PP in the follow-up scheme of patients with MEN1. In addition, both CgA and PP are susceptible to false positive results, as evidenced by the low specificity of biomarkers in the current study. Lung NETs are encountered in approximately 20% of patients with MEN1 [30], and are potentially responsible for elevated CgA. While some evidence suggests that SSTR PET/CT has good diagnostic capability in detecting lung NETs [31, 32], and our local surveillance protocols include thoracic CT at least every five years, we cannot rule out the possibility of undiagnosed lung NETs interfering with the CgA measurements. In addition, increased CgA concentrations are encountered in gastric and pancreatic cancer, prostate adenocarcinoma, and colorectal and hepatocellular cancer [33]. PPI treatment, impaired renal function and atrophic gastritis can also yield false positive CgA concentrations [33–36]. Furthermore, our study highlights the potential psychological burden and impaired quality of life, as suggested by van Leeuwaarde et al. [34], caused by clinically insignificant and false-positive results of biomarker screening during long term follow-up. Our study underscores the need for appropriate surveillance protocols in the follow-up of patients with MEN1.

Our study has some shortcomings. The use of different CgA measurement methods and ULNs over the years might reduce the reliability of our results. However, assays change over time reflecting the real-life long-term follow-up setting in MEN1. On the other hand, repeated measurements decrease the likelihood of between-assay variability. As the surgical removal of smaller panNETs is seldom appropriate, an optimal reference standard based on pathological samples was not possible. In addition, there was not enough power for both biomarkers to reach statistical significance for all considered diagnostic endpoints.

In conclusion, CgA and PP are not helpful in diagnosing panNETs in MEN1, not even in cases where tumor size exceeds 20 mm, the recommended cut-off for surgery. Neither was long-term biomarker screening over 10 years helpful. A proper screening approach is required in MEN1, and it is time to revise the surveillance protocol in practice.

## Data Availability

The data that support the findings of this study are not openly available due to privacy concerns and legislative restrictions on register studies. Further information is available from the corresponding author. Data are located in controlled access data storage at the Helsinki University Hospital.
